# Barriers to physical activity in pregnant women living in Iran and its predictors: a cross sectional study

**DOI:** 10.1186/s12884-022-05124-w

**Published:** 2022-11-04

**Authors:** Zari Dolatabadi, Leila Amiri-Farahani, Katayon Ahmadi, Sally Pezaro

**Affiliations:** 1 Student Research Committee, School of Nursing and Midwifery, Iran University of Medical Sciences, Tehran, Iran; 2 Nursing and Midwifery Care Research Center, Department of Reproductive Health and Midwifery, School of Nursing and Midwifery, Iran University of Medical Sciences, Tehran, 1996713883 Iran; 3 The University of Notre Dame, Fremantle, Australia; 4 The Centre for Healthcare Research, Coventry University, Coventry, UK

**Keywords:** Pregnancy, Physical activity, Barriers to physical activity

## Abstract

**Background and aims:**

Despite the benefits of physical activity (PA) on maternal and fetal health, the level of PA is low among pregnant women globally. The aim of this study was to determine the barriers to PA and its predictors in Iranian pregnant women specifically.

**Methods:**

This cross-sectional study included 300 pregnant women referred to the Ilam health centers of Iran. The sampling strategy used stratified random proportional allocation sampling from both comprehensive health centers and health bases. Data were collected from September to December 2018 in relation to individual characteristics. Data collection tools used included the Pregnancy Physical Activity Questionnaire and the Barriers to Physical Activity during Pregnancy Scale. To analyze the data, descriptive statistics and statistical tests of analysis including variance, independent t-test and multiple linear regression were used.

**Results:**

The mean and SD of the total score of PA barriers was 88.55 and 19.28, respectively. The highest and lowest mean scores of the subscale of PA barriers were related to interpersonal and environmental barriers, respectively. Among the intrapersonal barriers related to pregnancy; fear of pregnancy complications, drowsiness, and nausea and vomiting, heaviness or swelling barriers scored higher than other barriers. Lack of regular schedule, insufficient time, and lack of motivation received the highest score in terms of intrapersonal barriers non-related to pregnancy. In the interpersonal subscale; lack of knowledge about how to be physically active during pregnancy, forbiddance of PA by friends and family, as well as lack of advice from physicians and midwives scored higher than other barriers. Lack of adequate facilities and air pollution were identified as barriers to PA in the environmental subscale. PA barriers were significantly associated with pre-pregnancy or early pregnancy body mass index (B = − 14.643), level of education (B = 17.215), and habitual exercise pre-pregnancy (B = − 7.15).

**Conclusion:**

Interpersonal barriers were reported to be the most common barriers to PA during pregnancy. Perinatal care providers should encourage, educate and reassure pregnant women, their spouses and their families about the benefits, type and frequency of safe PA in pregnancy. PA interventions focused on women with lower levels of education and income in particular are required.

**Supplementary Information:**

The online version contains supplementary material available at 10.1186/s12884-022-05124-w.

## Introduction

Pregnancy is a unique period leading to numerous hormonal and physiological changes such as increases in blood volume, heart rate and weight [1]. Regular physical activity (PA) during pregnancy is associated with many benefits for the mother and the developing fetus [2]. For example, PA aids with the maintenance of a healthy weight during pregnancy, along with reducing the risk of gestational diabetes and preeclampsia [2, 3]. Active pregnant women also have lower rates of preterm labor [4], along with reduced rates of miscarriage and cesarean section and increased vaginal birth rates [5]. PA can also reduce postpartum depression [6, 7]. Moreover, PA during pregnancy can positively impact postnatal health and decrease the resulting child’s risk of developing chronic diseases such as obesity, diabetes, and CVD [8]. Despite the efficacy of PA during pregnancy and the development of guidelines for promoting PA, the majority of pregnant women have low adherence levels to PA guidelines during pregnancy, and many remain inactive during and after pregnancy [9].

The American College of Obstetricians and Gynecologists (ACOG) recommends moderate-intensity PA for women experiencing a healthy pregnancy for at least 150 minutes per week [10]. Other recommendations state that PA should be light to moderate and undertaken for approximately 120 to 150 minutes per week unless contraindicated [11]. Despite such recommendations, population data suggests that only 23 to 29% of pregnant women in the United States of America (USA) engage in sufficient PA during pregnancy [12]. The results of a separate study also demonstrated that only 31% of pregnant women in the Southeast of the USA engaged in low-intensity PA, whilst 38% engaged in moderate-intensity PA, and 32% engaged in high-intensity PA [13]. In the Ilam province of Iran, the highest levels of PA have been related to light PA (based on energy expenditure (≤ 2.9 MET- hour/week) whilst the lowest levels have been related to vigorous PA (> 6.0 MET- hour/week) [14]. Yet the barriers related to PA in Iranian pregnant women and its predictors have yet to be fully investigated. The lack of research in this population is concerning, particularly as other studies have demonstrated how women engaged in PA pre-pregnancy can reduce their level of PA after pregnancy [15, 16].

Pregnancy is an ideal time to adopt and maintain healthy lifestyle habits due to the mother’s interest in the child’s health [17]. However, women face many barriers to PA during pregnancy. Previous studies have identified these barriers as being related to intrapersonal, interpersonal, environmental, organizational, and political factors [18, 19]. Intrapersonal barriers to PA can include extreme fatigue, lack of time for exercise, and physical limitations such as joint pain, pelvic pain, edema, back pain and physical discomfort, and fear of harm to the unborn child [20]. Among the interpersonal barriers can be the negative reactions of others in relation to PA in pregnancy, and a lack of awareness about its benefits [18]. The high cost of sports and exercise classes has also been identified as a barrier in some cases among pregnant women in Benin, Nigeria [18].

A variety of factors are associated with increased PA in pregnancy [21]. In particular, being white, not having children, and engaging in PA pre-pregnancy have been associated with increased PA during pregnancy [22]. Nevertheless, pregnant women with younger age and lower family support may experience increased barriers to PA [23]. The barriers to PA in pregnancy and its predictors may be different for Iranian women in particular, as they demonstrate particularly low participation in moderate to vigorous PA [14]. Moreover, studies examining the barriers to PA in pregnancy which use validated tools remain absent. Consequently, the research team identified the need for a cross-sectional study in this field. The aim of this study was to determine the barriers of PA and its predictors in Iranian pregnant women.

## Methods

### Study design

The present cross-sectional study included pregnant women receiving perinatal care in both the health centers and health bases of Ilam city. Ilam is a city and capital of Ilam Province, Iran. At the 2017 census, its population was 194,030 people [24]. Health center bases are a subcategory of comprehensive urban health centers located in the city’s suburbs.

### Study sample

Our study sample and sampling strategy has been reported in our previous research [14]. Here, we used G * power software to determine the required sample size. The sample size calculation yielded a required number of 300 participants, based on a 95% confidence level, a power of 0.8, an effect size of 0.06 and the consideration of 11 predictor variables.

Recruitment occurred from September to December 2018. Comprehensive health centres (Larger healthcare organisations with a broad range of facilities and services) and health bases (Smaller with limited facilities and services) both include perinatal services providing prenatal clinical care. Stratified random proportional allocation sampling was used in that equal numbers of participants were recruited via both health centres and health bases in equal measure.

Participants were included if they were Iranian women, aged between 18 and 45 years, engaging in light PA during pregnancy, were between their 10th and 37th week of pregnancy, had no contraindications to exercise during pregnancy, had no movement restrictions, had the ability to read and write in Persian, and gave their informed written consent to participate in the study.

### Outcome measures and measurements

The survey invited responses in relation to participants’ individual characteristics (independent variables). Data collection tools also included the Pregnancy Physical Activity Questionnaire (PPAQ) [25, 26] and the Barriers to Physical Activity during Pregnancy Scale’ (BPAPS) [27] (dependent variable). All questions were completed via the self-reporting method by those who met the inclusion criteria.

Questions relating to individual characteristics consisted of variables such as age, pre-pregnancy, or early pregnancy body mass index (BMI), ethnicity, level of education, occupation, income (reported based on individual’s perception of income and to what extent it meets individual’s living needs), number of previous pregnancies, number of children, gestational age, participation in childbirth preparation classes and whether exercise was habitual before pregnancy.

The PPAQ designed by Chasan-Taber and colleagues assesses PA levels during current trimester of pregnancy [25]. This questionnaire asks respondents to select the category that best approximates the amount of time spent in 32 activities, including household/ caregiving, occupational, sports/exercise and inactivity during the current trimester. At the end of the PPAQ, an open-ended section allows the respondent to add additional activities not listed. The duration of time spent in each activity is multiplied by its intensity to measure average weekly energy expenditure (MET-hour/week) attributable to each activity. Finally, the activities are divided into seven categories: sedentary activity, light-intensity, moderate-intensity, vigorous-intensity, household/caregiving, occupational and sports/exercise. This questionnaire′s reliability has been confirmed via a Cronbach’s alpha of 0.78 for the total score and 0.87–0.93 for questionnaire categories among pregnant women at a large tertiary care facility in western Massachusetts, USA [25]. The validity and reliability of the Persian version of PPAQ were confirmed by Fathnezhad Kazemi and colleagues among pregnant women living in Tabriz, Iran [26].

The BPAPS designed by Amiri-Farahani and colleagues assesses barriers of exercise during pregnancy [27]. It includes 29 items, structured under four factors, including pregnancy-related intrapersonal barriers, non-pregnancy related intrapersonal barriers, interpersonal barriers, and environmental barriers [27]. Responses to the BPAPS are scored on a Likert 5-point scale as follows; 5 = strong agreement, 4 = agreement, 3 = neutral, 2 = disagreement, and 1 = strong disagreement. Based on the results obtained, the total score of BPAPS can range from 29 to 145 with a higher score associated with greater barriers to PA during pregnancy. Internal consistency and stability of the scale was confirmed by a Cronbach alpha coefficient of 0.824 and a test-retest reliability score of 0.87. With regard to the BPAPS′s reliability, the Cronbach’s alpha coefficients of the total scale and subscales of pregnancy-related intrapersonal barriers, non-pregnancy related intrapersonal barriers, interpersonal barriers, and environmental barriers were 0.82, 0.81, 0.73, 0.73 and 0.72, respectively [27]. Thus, the use of this tool alongside the PPAQ was considered appropriate in meeting the aims of the present study.

### Ethical approval

This study was approved by Ethics Committee of Iran University of Medical Sciences, Tehran, Iran (Number: IR.IUMS.REC.1397.1143). In addition, informed written consent was obtained from the participants, who were fully informed of the purpose and procedures of the study. Participants were also assured of confidentiality of information. All methods were carried out in accordance with our study protocol, along with relevant guidelines and regulation associated with the Iran University of Medical Sciences and professional regulatory bodies such as the Nursing and Midwifery Council.

### Analyses

The data were analysed using SPSS V.21 (SPSS). Following the assessment of skewness and kurtosis, the quantitative data were considered to be normally distributed. Descriptive statistics, including frequencies and percentages, mean and SD, were used for describing individual characteristic variables, along with barriers to PA. In relation to the subscales of the BPAPS, higher scores were considered indicative of a greater number of barriers. To calculate each subscale’s normalized score, its score was subtracted from the minimum score of that subscale and divided by the difference of maximum and a minimum score of that subscale. Finally, the answer obtained was multiplied by 100.

To compare the barriers of PA (quantitative variables) among individual characteristic variables (categorical variables), an independent t-test and ANOVA were used. Also, in this comparison, the partial eta square effect size, Cohen’s d effect size, and confidence interval were reported. According to Colin et al. (2012), partial eta square effect sizes are classified as small (0.01), medium (0.06), and large (0.14 and higher), [28]. Here, the effect sizes were reported based on Cohen’s d, and Standardized Mean Difference was reported based on Cohen’s d effect size (null effect = 0, trivial effect = 0–0.19, small effect = 0.2 = 0.49, medium effect = 0.5–0.79, large effect = 0.8–1.19, very large effect = 1.2–2, and huge effect ≥2), [29, 30]. To determine the relationship of each one of the independent variables (individual characteristic variables) on the dependent variable (barriers to PA separately), those variables that confirmed significance in the bivariate test (*p* < 0.05) were entered into a multiple linear regression model using a backward strategy. The backward strategy is a stepwise regression approach that begins with a full (saturated) model. At each step this strategy gradually eliminates variables from the regression model to find a reduced model that best explains the data. Before the multivariate analysis, regression assumptions, including normality of residuals, homogeneity of residual changes, and alignment of outliers and residuals independence were examined and confirmed. Results from the linear regression analysis are presented as beta coefficients with associated 95% CIs. The level of statistical significance was set at *p* < 0.05.

## Results

The mean age and SD of study participants was 27.52 and 5.28, respectively. Pre-pregnancy/early pregnancy (before 12 weeks) BMI was 26.03 ± 4.28 kg /m^2^. The mean and SD of gestational age was 23.77 and 8.61, respectively. The majority of participants had a university education (*n* = 181; 60.33%) and were considered fairly favorable in economic status (*n* = 150; 50%). Many participants (86%) identified themselves as being housewives and Kurdish. There were statistically significant relationships between the total score of PA barriers and pre-pregnancy/early pregnancy BMI (*p* < 0.001), education (*P* = 0.004), income (*p* < 0.001), number of pregnancies (*p* < 0.001) and habitual exercise pre-pregnancy (*p* < 0.001). As demonstrated in Table 1, these relationships were not statistically significant for variables relating to age, ethnicity, occupation, number of children, gestational age, and participation in childbirth preparation classes. Supplementary tables 1 to 4 show the relationship of pregnant women’s individual characteristics with the subscales of PA barriers.

**Table 1:** The relationship of pregnant women’s individual characteristics with the PA barriers

Variable		n	Mean	SD	***P*** value	^c^ES (CI^d^)
**Age**	≤ 24	85	89.91	16.88	^a^*P = *0.74	0.12 (− 0.11,0.37)
	25–29	98	86.87	18.42		
	30–34	92	88.78	21.84		
	≥ 35	25	89.68	20.87		
**Pre-pregnancy or early pregnancy BMI (kg/m2)**	≤ 18.5	12	88.83	14.14	^**a**^***P* < 0.001**	0.48 (0.24,0.72)
	18.5–24.9	107	88.36	19.44		
	25–29.9	132	88.17	19.09		
	≥ 30	49	89.93	20.93		
**Ethnicity**	Fars	11	84.9	7.24	^a^*P* = 0.12	0.19 (−0.44,0.79)
	Kurdish	256	88.02	19.65		
	Lur	24	97.21	19.21		
	Lak	9	85.11	13.96		
**Level of education**	Secondary	21	101.76	19.73	^**a**^***P* = 0.004**	−.074 (−1. 19,-0.3)
	Diploma	98	88.50	18.15		
	University education	181	87.05	19.35		
**Occupation**	Employed	41	91.37	17.71	^b^*P* = 0.325	0.169 (− 0.161, 0.499)
	Housewife	259	88.11	19.52		
**Income (millions Rls)**	Undesirable < 20	34	88.02	15.22	^**a**^***P*** **< 0.001**	0.44 (0.21,0.68)
	Fairly favorable: 20–40	150	92.67	18.19		
	Optimal: 40–100	116	83.38	20.55		
**No of pregnancies**	1	152	89.53	18.97	^**a**^***P*< 0.001**	−0.6 (−1.27,0.05)
	2	94	85.92	17.78		
	3	45	86.08	21.33		
	4	9	111.88	13.54		
**No of children**	0	153	89.43	18.92	^a^*P* = 0.102	−1.27 (−1.94,-0.6)
	1	112	85.88	18.52		
	2	35	93.28	22.37		
**Gestational age (weeks)**	10–14	67	85.67	15.83	^a^*P* = 0.22	0.13 (−0.09,0.37)
	15–28	123	90.62	21.45		
	29–37	110	88	18.52		
**Participation in childbirth preparation classes**	Yes	34	92.91	18.14	^b^*P* = 0.16	0.255 (− 0.103, 0.613)
	No	266	88	19.39		
**Habitual exercising pre-pregnancy**	Yes	110	79.59	20.95	^**b**^***P*< 0.001**	**0.783 (0.54, 1.027)**
	No	190	93.74	16.16		

Significance level: *P* < 0.05

^a^One-way ANOVA, ^b^Independent sample t-test, ^c^ Effect size, ^d^ Confidence interval

The mean and SD of the total score of PA barriers was 88.55 and 19.28. To compare the subscales of PA barriers, scores were calculated based on 100. In the present study, a higher score is indicative of greater barriers. As demonstrated in Table 2, the highest and lowest scores were related to interpersonal and environmental barriers. Items with a higher mean score were identified in the following subscales: intrapersonal barriers related to pregnancy; intrapersonal barriers non-related to pregnancy; interpersonal barriers; and environmental barriers. These related to the following items; “I cannot be physically active because of the heavy feeling of pregnancy (swelling and/or weight)”; “I cannot be physically active because I do not have a regular schedule in life”; the physician/midwife does not provide advice on how to do physical activity safely during pregnancy; and “there is too great a distance from my home to facilities designed for physical activity”, respectively.

**Table 2:** Scores of PA barriers and its Subscales (*n* = 300)

Subscale		Items	Mean	SD	Total Mean ± SD	Minimum	Maximum
**Intrapersonal barriers**	**Intrapersonal barriers related to pregnancy**	I cannot be physically active because of drowsiness.	3.04	1.35	29.47 ± 8.64 55.76 ± 20.58^a^	11	49
		I cannot be physically active because of lethargy/lack of energy.	2.50	1.30			
		I cannot be physically active because I do not have exercise habits.	2.84	1.30			
		Pregnancy is a time for rest	3.52	1.46			
		I cannot be physically active because of the heavy feeling of pregnancy (swelling and/or weight).	3.53	1.49			
		I cannot be physically active because of my abdominal size and appearance.	2.59	1.35			
		I cannot be physically active because of pain (such as back pain, hip pain, and/or headache).	2.70	1.33			
		I cannot be physically active because of shortness of breath.	2.61	1.38			
		I am concerned by possible pregnancy complications such as miscarriages and premature labor.	3.13	1.47			
		I cannot be physically active because of pregnancy gastrointestinal problems (such as nausea, vomiting, and heart burn).	3.04	1.36			
	**Intrapersonal barriers non-related to pregnancy**	Physical activity is too hard work for me.	2.74	1.28	15.52 ± 4.15 51.40 ± 23.73^a^	5	25
		I do not do physical activity because of a lack of confidence in my physical ability.	2.66	1.30			
		I do not have the patience to do physical activity.	3.39	1.32			
		I cannot be physically active because I do not have a regular schedule in life.	3.95	1.23			
		Because of family and childrearing responsibilities/activities I do not have enough time to do physical activity.	2.76	1.28			
**Interpersonal barriers**		In our society, it is not customary for pregnant women to do physical activity.	2.62	1.30	16.37 ± 4.59 57.07 ± 22.80^a^	5	25
		I do not do physical activity because I do not have access to complete information about physical activity during pregnancy.	3.41	1.43			
		My friends and relatives forbid me from doing physical activity during pregnancy.	3.14	1.38			
		The physician/midwife does not provide advice on the benefits of physical activity during pregnancy.	3.53	1.34			
		The physician/midwife does not provide advice on how to do physical activity safely during pregnancy	3.66	1.35			
**Environmental barriers**		Air pollution prevents me from doing physical activity outdoors.	3.49	1.34			
		I do not do physical activity because I do not have access to a suitable vehicle for transportation.	3.27	1.41	27.19 ± 6.55 50.52 ± 18.24^a^	9	45
		It is difficult for me to do physical activity in unfavorable weather (too cold/hot).	2.07	1.01			
		I am not able to pay for physical activities.	2.83	1.38			
		There are no specific physical activity programs designed for pregnant women.	2.78	1.35			
		Parks are unsafe and unsuitable for pregnant women to do physical activity.	2.50	1.28			
		I do not do physical activity because of a lack of space at home.	3.04	1.46			
		There is too great a distance from my home to facilities designed for physical activity.	3.95	1.25			
		There are very few places for me to do physical activity.	3.23	1.29			
**Total**					88.55 ± 19.28	36	133

^a^ Based on 100

To estimate the effect of each of the individual characteristic variables on barriers to PA, all variables with *P* < 0.05 based on the results of Table 1 were entered into the linear regression model using the backward method. The relationship of pregnant women’s individual characteristics with PA barriers is presented in Table 3, where some of the variables that entered into the model including pre-pregnancy/early pregnancy BMI, level of education, and habitual pre-pregnancy exercise remained in the model. As shown, for every one score decrease in the pre-pregnancy/early pregnancy BMI in the normal category, the barriers of the PA increased by 14.64 units. Women with a secondary education were reported to have a higher score (17.21 units higher) than those with a university education. The barriers score of PA in women who habitually exercised pre-pregnancy decreased by 7.15 units. Consequently, 13.9% of the variations in the dependent variable (Barriers of PA) were justified by the independent variables (pre-pregnancy/early pregnancy BMI, level of education, and the presence of habitual exercising before pregnancy).

**Table 3:** Relationship of pregnant women’s individual characteristics with PA barriers based on the results of multiple linear regression analysis

Independent variables		Unstandardized coefficients B	Standardized coefficient beta	95% CI for B	***P*** value	R2
**Pre –pregnancy/early pregnancy BMI (kg/m2)**	≤ 18.5	Reference category				0.139
	**18.5–24.9**	−14.643	−0.364	−25.991 to −3.295	**0.012**	
	**25–29.9**	−8.355	−0.216	−19.615 to 2.905	0.145	
	**≥ 30**	0.432	0.008	−11.872 to 12.737	0.945	
**Level of education**	**Secondary**	17.215	0.229	7.545 to 26.886	**0.001**	
	**Diploma**	5.242	0.128	−1.729 to 12.213	0.140	
	**University education**	Reference category				
**Income (millions Rls)**	**Undesirable < 20**	5.701	0.094	−1.317 to 12.719	0.111	
	**Fairly favorable:** **20–40**	0.541	0.014	−3.912 to 4.994	0.811	
	**Optimal: 40–100**	Reference category				
**No of pregnancies**		0.266	0.011	−2.419 to 2.951	0.846	
Habitual exercise before pregnancy		−7.150	−0.179	−13.994 to − 0.307	**0.041**	

## Discussion

The present study investigated the barriers of PA and its predictors in Iranian pregnant women. The mean and SD of total score of PA barriers were 88.55 and 19.28, respectively. The highest mean score was related to interpersonal barriers to PA in pregnancy. The lowest mean score was related to environmental barriers to PA in pregnancy.

In respect of intrapersonal barriers related to pregnancy, barriers predominantly related to fear of pregnancy complications such as miscarriage or preterm labor. Feelings of drowsiness, nausea, and vomiting, heaviness, or swelling were also identified as the most significant barriers to PA in pregnancy. Given the physiological changes which occur during pregnancy, this finding may not be surprising. Many participants also stated that pregnancy was a time to rest, rather than engage in PA. Previous studies conducted elsewhere have reported similar pregnancy-related intrapersonal barriers to PA such as drowsiness and fatigue, nausea and vomiting, and movement limitations due to weight gain [17, 20, 31–34]. Similarly, feelings of anxiety and fear of injury have been cited in several other studies as additional barriers to PA in pregnancy [17, 19, 35, 36]. This indicates a need for pregnancy care providers to educate and reassure pregnant women about safe PA in pregnancy. Appropriate interventions in accordance with the physical changes during pregnancy designed to maintain women’s participation in PA have also been suggested to assist in pursuit of increasing PA in pregnancy [19].

Regarding intrapersonal barriers non-related to pregnancy, lack of a regular schedule in life, lack of sufficient time, and lack of motivation to do PA scored the highest when compared to other barriers. Indeed, in other areas, women have also been found to have little opportunity to engage in PA during pregnancy due to various roles such as caring for children, working at home, and being employed [20]. Similarly, specific cultural characteristics among Iranian women typically reflect the principle that family responsibilities are prioritised and can thus prevent any sport and leisure activities [37, 38]. Supporting PA in the form of companionship during exercise and / or assistance with childcare and household chores by family members may be important to overcoming such barriers. Since one of the causes of decreased PA during pregnancy is also the fear of harm to the unborn child, education about the positive effects of PA in pregnancy may also be an important motivating factor for stimulating women to engage [39].

In the present study, the subscale of interpersonal barriers to PA received the highest score. In this regard, many researchers claim that interpersonal barriers can be the most important factors in women’s physical inactivity during pregnancy [20, 40, 41]. Among the interpersonal barriers, lack of knowledge about how to be physically active during pregnancy, prohibition of PA by friends and family, as well as lack of advice from physicians and midwives to perform PA during pregnancy received the highest scores. Previous studies have also reported how family and friends played an important role in women’s understanding of the perceived dangers of PA during pregnancy [14, 17, 41, 42]. In particular, the study by Harrison et al., reported how a lack of family support was significantly associated with greater barriers to PA during pregnancy [19]. Indeed, social support can be a key source of emotional and informational support for diet and physical activity-related beliefs and behaviors among pregnant women [43]. As such, physicians and midwives may play an important role in raising awareness of the benefits and importance of PA during pregnancy. This role is especially important given that pregnant women can receive conflicting information from family members on this topic [44]. Such education may also be tailored to involve families and spouses given that pregnant women cite support from their family, (particularly their partners) as being an important motivator for engaging in PA [45].

Among environmental factors, the majority of women cited the lack of adequate facilities for PA and air pollution as barriers to PA. These results emulate those of previous studies elsewhere [2, 34]. Indeed, access to facilities is one of the most important environmental factors of PA behavior in different communities [46]. Such access is increased for women with higher education and income [47]. Likewise in the present study, the total score of barriers to PA had a statistically significant relationship with individual characteristics such as women who had an increased pre-pregnancy/early pregnancy BMI, a lower level of education and those who reported habitual exercise before pregnancy. Barriers to PA in pregnancy similarly remain in overweight and obese pregnant women elsewhere, where knowledge around safe types of physical activity in pregnancy and awareness of the potential benefits of PA in pregnancy remain low [48]. Accordingly, efforts to increase PA should be focused on increasing equal access to safe and appropriate spaces for PA such as air-conditioned gymnasiums and sports centres for all and increasing awareness of the benefits of PA in pregnancy more widely.

Whilst results reported by Fell et al. are consistent with the present study [49], the results reported by Santos et al., demonstrate no statistically significant relationship between women’s pre-pregnancy BMI and barriers to PA [50]. Likewise, in other studies, no significant relationship was found between women’s pre-pregnancy BMI and exercise during pregnancy [21, 51, 52]. However, in the study of Baena-García et al., obesity in women was associated with increased PA [53]. Such findings point to inconsistencies in the literature. Therefore, further research is required to ensure all pregnant women, irrespective of BMI, have opportunity to engage in prenatal physical activity. Health experts specifically recommend increasing PA in overweight and obese women to promote continued PA during pregnancy [53–55].

Low income was significantly associated with scores relating to PA barriers. In previous studies, low income has likewise been identified as one of the most important barriers to PA during pregnancy [56, 57]. Contrariwise, high income levels have been an important predictor of continued exercise during pregnancy [22, 53, 58]. Nevertheless, it is important to consider that pregnant women with poor socioeconomic status may have to walk instead of using a car or even public transportation, and therefore spend more time walking during pregnancy [52, 59]. Increased pregnancy complications, as well as weight gain that can occur in conjunction with increased parity are also among the barriers to PA in many studies elsewhere [19, 60]. Further research will be required to explore how these particular barriers may be overcome in the context of Iran.

Habitual exercise prior to pregnancy had a statistically significant relationship with barriers to PA. Indeed, pre-pregnancy PA has been identified as one of the predictors of PA during pregnancy elsewhere [61]. Nevertheless, we found no statistically significant relationships in relation to the variables of age, ethnicity, occupation, number of children, gestational age, and participation in childbirth preparation classes. Similarly, no statistically significant relationships between age and barriers to PA have been found elsewhere [50, 62]. Yet women over the age of 35 have reportedly experienced greater barriers to PA in comparison to their younger counterparts [13]. In this study, different types of ethnicities were not significantly associated with barriers to PA. This is consistent with the results of an alternative study by DiPietro and colleagues [2]. Others have similarly shown no relationship between ethnicity in both active and inactive women during pregnancy [21]. Only one previous study has demonstrated a difference in the level of PA during pregnancy and ethnicity [63]. Future studies designed to collect qualitative data may be required to understand these discrepancies in more depth.

In our study, no statistically significant relationship was observed between employment status and barriers to PA [50]. This contradicts results presented elsewhere, where employment status was found to be one of the predictors of PA during pregnancy [56, 63]. In the present study, no relationship was found between the number of children and barriers to PA. This again contradicts results presented elsewhere, where the absence of children has been identified as an important predictor of exercise in pregnancy [22, 63, 64]. As such, there may be contextual factors consider in exploring this topic in future.

A key strength of this study is that it has included women from several centers in Iran and measured barriers to PA in all three trimesters of pregnancy using validated scales. Yet as only low-risk women were included in the sample, these results are not representative of all Iranian pregnant women. Future studies conducted with women experiencing low- risk pregnancies and/or high-risk pregnancies living in rural areas who can safely engage in PA are required. Another limitation of the study was the use of self-report measures, which may result in participants offering more socially acceptable (rather than sincere) answers [65]. Future studies conducted using more objective tools for the measurements of PA (e.g., pedometers) are required. Studies designed to collect and analyze qualitative data are also required, both to complement these quantitative findings and provide context as to why and how such barriers to PA may present themselves in practice.

## Conclusion

The results of our study demonstrate that pregnant women in Iran face various barriers to PA during pregnancy. These lead to a decrease in PA during pregnancy. Raised awareness and education in relation to the benefits of PA during pregnancy and more support from family and spouses may increase PA during pregnancy. PA interventions including increased access to sports facilities and gymnasiums need to be targeted toward those with lower levels of education and income whose PA levels are low.

## Supplementary Information

**Additional file 1.**

## Data Availability

The datasets generated and analyzed during the current study are not publicly available due to the confidentiality of information, but they can be available through the corresponding author on reasonable request.
