# Supplementary material for: Barriers to physical activity in pregnant women living in Iran and its predictors: a cross sectional study
Source: BMC Pregnancy Childbirth. 2022 Nov 4;22:815. doi: 10.1186/s12884-022-05124-w (PMC9636628; doi:10.1186/s12884-022-05124-w)
Supplement: Supplementary file 1 — Additional file 1. [file 12884_2022_5124_MOESM1_ESM.docx]

**SUPPLEMENTARY TABLE 1. The Relationship of pregnant women’s individual characteristics with intrapersonal barriers related to pregnancy**

| **Variable** | | **n** | **Mean** | **SD** | **P value** | *****ES (CI****)** |
| --- | --- | --- | --- | --- | --- | --- |
| **Age** | 24 ≥ | 85 | 30.50 | 8.01 | 0.07 = *P | 0.25  (0.004,0.5) |
|  | 25-29 | 98 | 27.84 | 7.78 |  |  |
|  | 30-34 | 92 | 29.55 | 9.14 |  |  |
|  | 35 ≤ | 25 | 31.96 | 11.14 |  |  |
| **Pre-pregnancy or early pregnancy BMI (kg/m2)** | 18.5 ≥ | 12 | 28.83 | 8.74 | 0.61 = *P | 0.343  (0.09,0.59) |
|  | 18.5-24.9 | 107 | 30.19 | 8.56 |  |  |
|  | 25-29.9 | 132 | 29.39 | 8.89 |  |  |
|  | 30 ≤ | 49 | 28.24 | 8.20 |  |  |
| **Ethnicity** | Fars | 11 | 27.63 | 5.62 | 0.16 =*P | 0.32  (-0.34,0.98) |
|  | Kurdish | 256 | 29.32 | 8.52 |  |  |
|  | Lur | 24 | 32.83 | 8.08 |  |  |
|  | Lak | 9 | 26.77 | 14.25 |  |  |
| **Level of education** | Secondary | 21 | 33.72 | 7.92 | **0.05 = *P** | -0.53  (-0.97,-0.08) |
|  | Diploma | 98 | 29.68 | 8.15 |  |  |
|  | University education | 181 | 28.86 | 8.88 |  |  |
| **Occupation** | Employed | 41 | 30.41 | 10.78 | 0.53 = **P | 0.126 (0.204, 0.456) |
|  | Housewife | 259 | 29.32 | 8.27 |  |  |
| **Income (milions Rls)** | Undesirable <20 | 34 | 26.52 | 7.36 | **0.001 *P <** | -0.44  (-0.67,-0.21) |
|  | Fairly favorable:  20–40 | 150 | 31.36 | 8.99 |  |  |
|  | Optimal: 40–100 | 116 | 27.88 | 8.02 |  |  |
| **No of pregnancies** | 1 | 152 | 29.05 | 8.08 | **0.003 = P*** | -1.26  (-1.93,-.59) |
|  | 2 | 94 | 29.11 | 8.93 |  |  |
|  | 3 | 45 | 29.51 | 9.24 |  |  |
|  | 4 | 9 | 39.88 | 5.88 |  |  |
| **No of children** | 0 | 153 | 29.19 | 8.10 | 0**.009 = P*** | -0.54  (-0.89,-0.18) |
|  | 1 | 112 | 28.56 | 8.90 |  |  |
|  | 2 | 35 | 33.57 | 9.20 |  |  |
| **Gestational age (weeks)** | 10-14 | 67 | 27.13 | 6.17 | **0.002 = *P** | -0.39  (-0.62,-0.16) |
|  | 15-28 | 123 | 31.43 | 8.78 |  |  |
|  | 29-37 | 110 | 28.69 | 9.34 |  |  |
| **Participation in childbirth preparation classes** | Yes | 34 | 29.17 | 9.12 | 0.83 = ** P | 0.038 (- 0.319, 0.395) |
|  | No | 266 | 29.5 | 8.60 |  |  |
| **Habitual exercising pre-pregnancy** | Yes | 110 | 24.14 | 8.18 | **0.001 < **P** | **1.1 (0.849, 1.351)** |
|  | No | 190 | 32.55 | 7.32 |  |  |

Significance level: P < 0.05

*One-way ANOVA, **Independent sample t-test, *** Effect size, **** Confidence interval

**SUPPLEMENTARY TABLE 2. The Relationship of pregnant women’s individual characteristics with intrapersonal barriers non-related to pregnancy**

| **Variable** | | **n** | **Mean** | **SD** | **P value** | *****ES (CI****)** |
| --- | --- | --- | --- | --- | --- | --- |
| **Age** | 24 ≥ | 85 | 15.88 | 4.26 | 0.11 = *P | 0.43  (0.02,0.84) |
|  | 25-29 | 98 | 15.24 | 3.86 |  |  |
|  | 30-34 | 92 | 15.92 | 4.37 |  |  |
|  | 35 ≤ | 25 | 13.88 | 3.72 |  |  |
| **Pre-pregnancy or early pregnancy BMI (kg/m2)** | 18.5 ≥ | 12 | 13.91 | 4.20 | 0.41 = *P | 0.39  (015,0.63) |
|  | 18.5-24.9 | 107 | 15.43 | 4.06 |  |  |
|  | 25-29.9 | 132 | 15.84 | 3.95 |  |  |
|  | 30 ≤ | 49 | 15.22 | 4.81 |  |  |
| **Ethnicity** | Fars | 11 | 15.63 | 2.57 | **0.04 =*P** | 0.52  (0.16,0.89) |
|  | Kurdish | 256 | 15.26 | 4.15 |  |  |
|  | Lur | 24 | 17.58 | 4.64 |  |  |
|  | Lak | 9 | 17.11 | 1.83 |  |  |
| **Level of education** | Secondary | 21 | 18.42 | 4.24 | **0.01 = *P** | -0.76  (-1.21,-0.32) |
|  | Diploma | 98 | 15.80 | 4.04 |  |  |
|  | University education | 181 | 15.02 | 4.06 |  |  |
| **Occupation** | Employed | 41 | 15.78 | 3.47 | 0.61 = **P | 0.072 (- 0.257, 0.402) |
|  | Housewife | 259 | 15.47 | 4.25 |  |  |
| **Income (milions Rls)** | Undesirable <20 | 34 | 14.35 | 3.24 | 0.14 = P* | 0.55  (-0.05,1.15) |
|  | Fairly favorable:  20–40 | 150 | 15.87 | 4.27 |  |  |
|  | Optimal: 40–100 | 116 | 15.40 | 4.18 |  |  |
| **No of pregnancies** | 1 | 152 | 15.78 | 3.94 | **0.004 = P*** | -1.04  (-1.71,-0.37) |
|  | 2 | 94 | 14.70 | 3.68 |  |  |
|  | 3 | 45 | 15.51 | 5.23 |  |  |
|  | 4 | 9 | 19.66 | 3.60 |  |  |
| **No of children** | 0 | 153 | 15.70 | 3.91 | **0.001 = P*** | -0.46  (-0.81,-0.1) |
|  | 1 | 112 | 14.46 | 3.91 |  |  |
|  | 2 | 35 | 18.08 | 4.73 |  |  |
| **Gestational age (weeks)** | 10-14 | 67 | 14.74 | 3.75 | 0.15 = *P | -0.37  (-0.64,-0.11) |
|  | 15-28 | 123 | 15.52 | 4.65 |  |  |
|  | 29-37 | 110 | 15.99 | 3.73 |  |  |
| **Participation in childbirth preparation classes** | Yes | 34 | 16.85 | 4.05 | 0.05 = ** P | 0.366 (0.0007, 0.724) |
|  | No | 266 | 15.34 | 4.14 |  |  |
| **Habitual exercising pre-pregnancy** | Yes | 110 | 15.44 | 5.01 | 0.82 = ** P | 0.029 (- 0.264, 0206) |
|  | No | 190 | 15.56 | 3.57 |  |  |

Significance level: P < 0.05

*One-way ANOVA,**Independent sample t-test, *** Effect size, **** Confidence interval

**SUPPLEMENTARY TABLE 3. The Relationship of pregnant women’s individual characteristics with interpersonal barriers**

| **Variable** | | **n** | **Mean** | **SD** | **P value** | *****ES (CI****)** |
| --- | --- | --- | --- | --- | --- | --- |
| **Age** | 24 ≥ | 85 | 16.10 | 3.76 | 0.89 = *P | 0.07  (-0.33,0.47) |
|  | 25-29 | 98 | 16.54 | 4.95 |  |  |
|  | 30-34 | 92 | 16.53 | 4.81 |  |  |
|  | 35 ≤ | 25 | 16.08 | 5.08 |  |  |
| **Pre-pregnancy or early pregnancy BMI (kg/m2)** | 18.5 ≥ | 12 | 17.41 | 3.34 | 0.05 = *P | -0.28  (-0.58,0.02) |
|  | 18.5-24.9 | 107 | 16.84 | 4.42 |  |  |
|  | 25-29.9 | 132 | 16.50 | 4.66 |  |  |
|  | 30 ≤ | 49 | 14.77 | 4.82 |  |  |
| **Ethnicity** | Fars | 11 | 16 | 2.48 | 0.57 =*P | 0.23  (-0.43,0.89) |
|  | Kurdish | 256 | 16.32 | 4.80 |  |  |
|  | Lur | 24 | 17.50 | 3.45 |  |  |
|  | Lak | 9 | 15.33 | 2.54 |  |  |
| **Level of education** | Secondary | 21 | 17.71 | 2.98 | **0**.37 = *P | -0.31  (-0.75,0.13) |
|  | Diploma | 98 | 16.36 | 4.51 |  |  |
|  | University education | 181 | 16.22 | 4.79 |  |  |
| **Occupation** | Employed | 41 | 16.47 | 3.02 | 0.81 = **P | 0.024 ( - 0.306, 0.353) |
|  | Housewife | 259 | 16.36 | 4.80 |  |  |
| **Income (milions Rls)** | Undesirable <20 | 34 | 16.35 | 4.28 | **0.01 =** P* | -0.33  (-0.56,-0.09) |
|  | Fairly favorable:  20–40 | 150 | 17.10 | 4.55 |  |  |
|  | Optimal: 40–100 | 116 | 15.44 | 4.61 |  |  |
| **No of pregnancies** | 1 | 152 | 16.83 | 4.13 | **0.02 = P*** | 0.42  (0.11,0.74) |
|  | 2 | 94 | 16.06 | 4.89 |  |  |
|  | 3 | 45 | 14.93 | 5.25 |  |  |
|  | 4 | 9 | 19.11 | 3.55 |  |  |
| **No of children** | 0 | 153 | 16.60 | 4.44 | **0.13 = P*** | 0.36  (0.007,0.71) |
|  | 1 | 112 | 16.51 | 4.80 |  |  |
|  | 2 | 35 | 14.91 | 4.46 |  |  |
| **Gestational age (weeks)** | 10-14 | 67 | 16.46 | 4.56 | 0.75 = *P | 0.08  (-0.14,0.31) |
|  | 15-28 | 123 | 16.13 | 5.06 |  |  |
|  | 29-37 | 110 | 16.59 | 4.07 |  |  |
| **Participation in childbirth preparation classes** | Yes | 34 | 17.67 | 3.77 | 0.07 = ** P | 0.319 (- 0.039, 0.677) |
|  | No | 266 | 16.21 | 4.67 |  |  |
| **Habitual exercising pre-pregnancy** | Yes | 110 | 14.20 | 4.65 | **0.001 = **** P | **0.799 ( 0.556, 1.043)** |
|  | No | 190 | 17.63 | 4.07 |  |  |

Significance level: P < 0.05

*One-way ANOVA,**Independent sample t-test, *** Effect size, **** Confidence interval

**SUPPLEMENTARY TABLE 4. The Relationship of pregnant women’s individual characteristics with environmental barriers**

| **Variable** | | **n** | **Mean** | **SD** | **P value** | *****ES (CI****)** |
| --- | --- | --- | --- | --- | --- | --- |
| **Age** | 24 ≥ | 85 | 27.42 | 6.08 | 0.87 = *P | 0.09  (-0.15,0.33) |
|  | 25-29 | 98 | 27.24 | 6.74 |  |  |
|  | 30-34 | 92 | 26.76 | 7.43 |  |  |
|  | 35 ≤ | 25 | 27.76 | 3.36 |  |  |
| **Pre-pregnancy or early pregnancy BMI (kg/m2)** | 18.5 ≥ | 12 | 28.58 | 4.60 | 0.41 = *P | -0.58  (-1.15,-0.004) |
|  | 18.5-24.9 | 107 | 27.36 | 6.71 |  |  |
|  | 25-29.9 | 132 | 27.42 | 6.27 |  |  |
|  | 30 ≤ | 49 | 25.83 | 7.30 |  |  |
| **Ethnicity** | Fars | 11 | 25.63 | 2.37 | 0.32 =*P | -0.34  (-0.76, 0.06) |
|  | Kurdish | 256 | 27.10 | 6.64 |  |  |
|  | Lur | 24 | 29.29 | 7.23 |  |  |
|  | Lak | 9 | 25.88 | 4.98 |  |  |
| **Level of education** | Secondary | 21 | 31.90 | 8.59 | **0.003 = *P** | -0.78  (-1.23, -0.33) |
|  | Diploma | 98 | 26.64 | 5.39 |  |  |
|  | University education | 181 | 26.93 | 6.69 |  |  |
| **Occupation** | Employed | 41 | 28.63 | 5.97 | 0.10 = **P | 0.255  ) - 0.075, 0.586) |
|  | Housewife | 259 | 26.96 | 6.62 |  |  |
| **Income (milions Rls)** | Undesirable <20 | 34 | 30.79 | 5.85 | **0.001 = P*** | -0.65  (-1.01, -0.29) |
|  | Fairly favorable:  20–40 | 150 | 28.34 | 5.81 |  |  |
|  | Optimal: 40–100 | 116 | 24.64 | 6.79 |  |  |
| **No of pregnancies** | 1 | 152 | 27.85 | 7.63 | **0.004 = P*** | -0.96  (-1.63, -0.29) |
|  | 2 | 94 | 26.04 | 4.30 |  |  |
|  | 3 | 45 | 26.13 | 6.18 |  |  |
|  | 4 | 9 | 33.22 | 3.07 |  |  |
| **No of children** | 0 | 153 | 27.92 | 7.25 | **0**.13 = P* | -0.22  (-0.045, -0.001) |
|  | 1 | 112 | 26.33 | 5.19 |  |  |
|  | 2 | 35 | 26.71 | 7.04 |  |  |
| **Gestational age (weeks)** | 10-14 | 67 | 27.32 | 5.20 | 0.63 = *P | 0.11  (-0.12,0.34) |
|  | 15-28 | 123 | 27.52 | 7.31 |  |  |
|  | 29-37 | 110 | 26.72 | 6.42 |  |  |
| **Participation in childbirth preparation classes** | Yes | 34 | 29.20 | 7.26 | 0.09 = ** P | 0.35 (- 0.008, 0.708) |
|  | No | 266 | 26.92 | 6.42 |  |  |
| **Habitual exercising pre-pregnancy** | Yes | 110 | 25.80 | 7.45 | **0.009 = ** P** | **0.338 (0.102, 0.574)** |
|  | No | 190 | 27.99 | 5.85 |  |  |

Significance level: P < 0.05

*One-way ANOVA, **Independent sample t-test, *** Effect size, **** Confidence interval
